# Hypoxic Preconditioning Differentially Affects GABAergic and Glutamatergic Neuronal Cells in the Injured Cerebellum of the Neonatal Rat

**DOI:** 10.1371/journal.pone.0102056

**Published:** 2014-07-17

**Authors:** Sergio G. Benitez, Analía E. Castro, Sean I. Patterson, Estela M. Muñoz, Alicia M. Seltzer

**Affiliations:** 1 Laboratory of Neurobiology: Chronobiology Section, Institute of Histology and Embryology of Mendoza (IHEM), School of Medicine, National University of Cuyo, Mendoza, National Scientific and Technical Research Council (CONICET), National Agency for Scientific and Technological Promotion (ANPCyT), Mendoza, Argentina; 2 Traumatic and Toxic Lesions in the Nervous System Section, Institute of Histology and Embryology of Mendoza (IHEM), School of Medicine, National University of Cuyo, Mendoza, National Scientific and Technical Research Council (CONICET), National Agency for Scientific and Technological Promotion (ANPCyT), Mendoza, Argentina; 3 Neonatal Brain Development Section, Institute of Histology and Embryology of Mendoza (IHEM), School of Medicine, National University of Cuyo, Mendoza, National Scientific and Technical Research Council (CONICET), National Agency for Scientific and Technological Promotion (ANPCyT), Mendoza, Argentina; National University of Singapore, Singapore

## Abstract

In this study we examined cerebellar alterations in a neonatal rat model of hypoxic-ischemic brain injury with or without hypoxic preconditioning (Pc). Between postnatal days 7 and 15, the cerebellum is still undergoing intense cellular proliferation, differentiation and migration, dendritogenesis and synaptogenesis. The expression of glutamate decarboxylase 1 (GAD67) and the differentiation factor NeuroD1 were examined as markers of Purkinje and granule cells, respectively. We applied quantitative immunohistochemistry to sagittal cerebellar slices, and Western blot analysis of whole cerebella obtained from control (C) rats and rats submitted to Pc, hypoxia-ischemia (L) and a combination of both treatments (PcL). We found that either hypoxia-ischemia or Pc perturbed the granule cells in the posterior lobes, affecting their migration and final placement in the internal granular layer. These effects were partially attenuated when the Pc was delivered prior to the hypoxia-ischemia. Interestingly, whole nuclear NeuroD1 levels in Pc animals were comparable to those in the C rats. However, a subset of Purkinje cells that were severely affected by the hypoxic-ischemic insult—showing signs of neuronal distress at the levels of the nucleus, cytoplasm and dendritic arborization—were not protected by Pc. A monoclonal antibody specific for GAD67 revealed a three-band pattern in cytoplasmic extracts from whole P15 cerebella. A ∼110 kDa band, interpreted as a potential homodimer of a truncated form of GAD67, was reduced in Pc and L groups while its levels were close to the control animals in PcL rats. Additionally we demonstrated differential glial responses depending on the treatment, including astrogliosis in hypoxiated cerebella and a selective effect of hypoxia-ischemia on the vimentin-immunolabeled intermediate filaments of the Bergmann glia. Thus, while both glutamatergic and GABAergic cerebellar neurons are compromised by the hypoxic-ischemic insult, the former are protected by a preconditioning hypoxia while the latter are not.

## Introduction

In recent years pediatricians have warned of a previously underappreciated injury to the cerebellum in extremely premature infants [1] and in those suffering from birth asphyxia [2]. Prematurity and/or hypoxia-ischemia in the perinatal period are among the risk factors underlying cerebral palsy, a condition known to derive from developmental disturbances to the immature brain that lead to substantial motor, cognitive, and learning deficits.

The pathophysiology of perinatal encephalopathy is difficult to study in the human, thus the neonatal rat model of hypoxic-ischemic brain injury has been useful in the analysis of this condition, considering that human brain development in late gestation seems to be equivalent to the immediate postnatal stage in rats and mice. The intensive application of the Rice and Vannucci model [3] of hypoxia-ischemia in the 7-day old rat has established several classic concepts with regards to immature brain vulnerability to selective neuronal death in major brain structures.

Defective brain development or damage to motor areas during critical periods of organogenesis further disrupts the brain's ability to adequately control movement and posture [4]. In the preterm/term fetus, umbilical cord compression often results in nearly complete asphyxia, leading to preferential injury to the peri-Rolandic cortex, putamen and thalamus [5]. More recently, magnetic resonance imaging (MRI) studies have provided a complete overview of the effects of severe hypoxia-ischemia in both preterm and term neonates. Deep gray matter (GM) injury with peri-Rolandic involvement is more frequently observed in the older age group. Less profound insults result in intraventricular hemorrhages and periventricular white matter (WM) injury in preterm neonates and parasagittal watershed territory infarcts in term neonates [6]; [7]. In the postnatal period (P), severe insults result in diffuse GM injury, with relative sparing of the peri-Rolandic cortex and the structures supplied by the posterior circulation. Profound hypoxia-ischemia in older children and adults affects the GM deep nuclei, cerebral cortices, hippocampi and cerebellum [8]–[10]. Thus, many critical neuronal populations at different maturation stages are at great risk during ischemic insults, for example projection neurons in the deep nuclei of the brainstem [11]; [12]. Peng et al. [13] pointed out that following focal cerebral hypoxic-ischemic injury, neuronal apoptosis accompanying necrosis occurs in the cerebellum, an area outside the vascular supply of the relevant ipsilateral hemisphere [10]. Later on it was found that hypoxia-ischemia at P2, the rat equivalent of human prematurity, cause damage to a subset of Purkinje cells, a significant decrease in the number of interneurons and in the thickness of molecular and granular layers [14]. The neurons of the anterior cerebellum (Lobes III–IV), which are less mature than the ones located in the posterior cerebellum (Lobes VIII and IX) [15], showed higher vulnerability. Therefore, in order to increase our understanding of the sequelae of asphyxia in the human perinatal period when the cerebellum is still developing, it is important to characterize in detail the effects in animal models.

The cerebellum is derived from the neuroepithelium that surrounds the lateral recess of the IV^th^ ventricle in the pons and the medulla, and its formation spans embryonic and postnatal development [16]–[19]. Despite its morphological and functional complexity, the on-going developmental processes that take place in the rat postnatal period make cerebellum an attractive and accessible model for perinatal hypoxia-ischemia. Several classes of finely interconnected neurons located in the cortex and deep nuclei contribute to cerebellar complexity. The glutamatergic granule cells and the GABAergic Purkinje cells represent the two main cell types analyzed in this study. The granule cells are important cerebellar interneurons that originate in the cortical external germinative layer (EGL), which derives from a neurogenic zone called the rhombic lip [20]–[22]. In the rat EGL, granule cell neurogenesis extends from P4 to P19 with a maximum between P8 and P15 [16]. Thus proliferation, radial migration into the cerebellar cortex along the processes of the Bergmann glia cells, differentiation, and settling mechanisms that give rise to the granule cells all take place during the period of the model of brain injury and/or hypoxic preconditioning described here (P7/8-P15). The time course of human cerebellar granule cell origin is less well defined, starting in the fetal period and ending in a variable range from the 7^th^ postnatal month to the end of the 2^nd^ year [16]. The Purkinje cells, the cerebellar output neurons with axons that project out of the cortex mainly towards the deep cerebellar nuclei, originate from a germinal niche in the roof of the IV^th^ ventricle much earlier than the granule cells, with a neurogenic peak at E15 in rat and around the 6^th^ week after fertilization in human [16]; [17]; [23].

Despite the neurogenic gap between granule and Purkinje cell populations, it has been shown that the terminal differentiation of rat Purkinje cells during the 2^nd^ and 3^rd^ weeks after birth is highly dependent on interactions with the Bergmann glia and cerebellar interneurons, including the granule cells and their parallel fibers [24]–[26]. This late stage in Purkinje cell differentiation involves specific features of dendritic arborization and orientation, amongst others. Two molecular markers have been chosen in this study to evaluate the effects of global and/or ischemic hypoxia on the developing cerebellum, a transcriptional factor known as NeuroD1/BETA2 (Neurogenic differentiation factor 1/β-cell E-box transactivator 2) [27]; [28] and the GABAergic enzyme GAD67 (Glutamate decarboxylase 1) [29].

The pro-neuronal role of NeuroD1 was first reported in *Xenopus* after ectopic expression that leads to neuron formation from the ectoderm [27]. NeuroD1 expression correlates well with granule cell differentiation in the cerebellum, showing stable levels in the internal granular layer (IGL) until adulthood [30]; [31]. Despite a near uniform cytoarchitecture, some genes are differentially expressed among the cerebellar lobes [32]; [33]. Preferential posterior cerebellum defects in granule cells were reported in the global absence of *NeuroD1* [30]; [34]. A conditional granule cell precursor-selective *NeuroD1* knock-out model resulted in elimination of the granule cells in the central lobes and anomalies in the Purkinje cells [35]. Although these results vary, depending on when and where *NeuroD1* is deleted during the cerebellar development, the genetically modified mice support the interactions between Purkinje and granule cells during the acquisition and maintenance of their final phenotypes.

GAD67 catalyzes the conversion of L-glutamate into γ–aminobutyric acid (GABA), the principal inhibitory neurotransmitter that can also exercise an excitatory influence in the immature brain, and on hippocampal neuronal precursors promoting adult neurogenesis [29]; [36]–[38]. GAD67 is encoded by a single gene, distinct from *Gad65* [29]. Different protein forms derived from alternative splicing of GAD67 mRNAs and with specific developmental expression patterns have been reported [39]; [40]. The transcription factor Egr1, a known regulator of genes associated with synaptic plasticity and memory [41], was also found to induce *Gad67* expression in hippocampal neurons [42]. Roybon et al. [43] proposed that basic helix-loop-helix (bHLH) transcription factors influence GABAergic or glutamatergic neuronal differentiation in a compensatory and cross-regulatory manner. Specifically these authors showed that NeuroD1, a downstream effector of neurogenins, can abrogate the GABAergic phenotype directed by Mash1 facilitating the glutamatergic fate. A direct inhibitory role of NeuroD1 on *Gad67* was not ruled out.

It is well known that neurogenesis and cell proliferation increase in the neonatal brain in response to ischemic injury and that multiple brain areas function as reservoirs of brain precursor cells [44]. The contribution of progenitor pools to recovery in the immature brain, which displays a window of plasticity, has been addressed in other reports [45]. In addition, immunocytochemical and electrophysiological studies have demonstrated that hypoxic preconditioning promotes neural progenitor differentiation and enhances cell survival [46]. Preconditioning is a phenomenon that promotes neuroprotective endogenous mechanisms that prevent future injury by adapting the tissues to low doses of noxious insults. Although not fully elucidated, it is believed that activation of the cell genome and protein synthesis is the general mechanism for inducing brain tolerance by hypoxic preconditioning. Molecules proposed to trigger and sustain the survival, differentiation and plasticity pathways include neuromodulatory peptides (e.g. Bcl-2); growth factors (e.g. IGF-1, BDNF and NGF); as well as the oxygen-sensitive transcription factors HIF-1a and HIF-2a [47].

Understanding how the neonatal brain reacts to injury and which endogenous strategies allow repair and survival is of utmost importance in devising effective therapeutic strategies. Here, we examine whether different hypoxic treatments affect cerebellar development, mainly by studying the expression of NeuroD1 and GAD67 as glutamatergic and GABAergic markers of granule and Purkinje cells, respectively.

## Materials and Methods

### Animals

We used Wistar Kyoto rat pups of either sex bred at our local animal facility. Their mothers were kept under controlled light–dark cycle (L:D 14:10) and temperature (23°C±1). The litters were allowed to nurse *ad libitum.* Each dam and her own litter were housed in individual metal cages with free access to food and water, and bedding was replaced periodically to keep it clean and dry. A total of 35 rat pups were studied in this project distributed into the following groups: L (hypoxia-ischemia) n = 12; Pc (hypoxic preconditioning) n = 6; PcL (hypoxic preconditioning plus hypoxia-ischemia) n = 9 and C (control) n = 8. The procedures performed on the animals were carried out in accordance with the regulations of the National Institutes of Health Guide for the Care and Use of Laboratory Animals (Eighth Edition, National Academy of Sciences, 2011), and approved by our Institutional Animal Care and Use Committee (CICUAL). Our Institution has PHS Approved Animal Welfare Assurance (Registry # A5780-01) and the experiments were described here according to ARRIVE guidelines.

### Induction of cerebral ischemic-hypoxic injury

At postnatal day 8 (P8), L animals were anaesthetized with sevofluorane and their right common carotid artery was dissected, permanently ligated at two points with 7-0 surgical silk and subsequently cut between both ligatures [48]. After two hours recovery from the anaesthesia in the original cage with the rest of the litters and dam, pups were exposed individually to hypoxia inside a controlled atmosphere chamber that was saturated with N_2_ (100%) and maintained at a temperature of 37°C by means of a water bath. Pups remained inside the chamber for 2–3 minutes, until they showed signs of asphyxia. After recovery of normal breathing, they were returned to their original housing. Pc animals were submitted to the preconditioning procedure described below on P7, while the PcL group was preconditioned on P7 and additionally received the same treatment as L pups on P8. The C pups were separated from their mothers for the same time periods as their surgery-exposed litter-mates. All animals were sacrificed at P15 by decapitation or deep anaesthesia according to the destination of the tissue, Western Blot (WB) or immunohistochemistry (IHC), respectively. All efforts were made to minimize suffering. In agreement with previous observations, this model of neonatal brain injury caused a variable degree of damage ranging from none to severe atrophy with cavitation [49]. In our hands, pup mortality was less than 1% [48]. In addition, 6.6% of L and 1.6% of PcL animals showed brain edema or cavitation at the time of sacrifice and these animals were excluded from this study.

### Induction of hypoxic preconditioning (Pc)

At P7, pups assigned to the Pc group were placed in 50 ml Falcon tubes with sealed caps until they evinced apnea (at least three consecutive mouth openings; usually about 25–30 minutes), and then taken out of the tubes. They were then allowed to breathe room air for 15 minutes. The whole procedure was repeated three times [48]. The pups were kept warm by means of a heating lamp, to avoid protection by cooling [50]. Twenty four hours after the preconditioning procedure, the pups belonging to the PcL group underwent hypoxia-ischemia as described above.

### Immunohistochemistry

Animals were deeply anaesthetized with ketamine/xylazine (50 mg/kg and 5 mg/kg body weight, respectively), then perfused intracardially with washing solution (0.8% sucrose and 0.4% glucose in PBS) followed by 4% paraformaldehyde in PBS pH 7.3. The whole brains were collected and post-fixed in the same paraformaldehyde mixture overnight. After fixation, the brains were washed three times in PBS, dehydrated in increasing concentrations of ethanol (70, 80, 96 and 100%), washed twice in xylene, and finally included in Histoplast (Biopack). Incubation times in the different solutions were around 60 minutes except during the last step in absolute alcohol which was performed overnight. Ten micrometer sagittal sections of the left side of the brain, including the cerebellum, were cut using a Microm HM-325 microtome. All the immunohistochemical procedures were performed as previously described [51]. Briefly, the brain sections mounted on positive charged slides were boiled in 0.1 M citrate buffer (pH 6) for 30 minutes in a pressure cooker for antigen retrieval and then washed three times in PBS for 5 minutes each. Non-specific labeling was blocked by incubating the brain sections in blocking solution (50 mM Tris–HCl pH 7.3, 0.125 M NaCl, 5% (v/v) normal horse serum-Vector Laboratories Inc., 20 mg/ml bovine serum albumin fraction V-Gibco BRL, and 5 mg/ml low-fat milk powder) for 30 minutes at room temperature in a covered humid chamber. Immunodetection was performed using the following primary antibodies diluted in the blocking solution without normal horse serum: goat anti-NeuroD1, sc-1084 (N19), Santa Cruz Biotechnology, dilution 1∶25; mouse monoclonal anti-glutamate decarboxylase 1 (GAD67), ab26116 (K-87), Abcam, dilution 1∶200; mouse monoclonal anti-vimentin (VIM), V-6630, Sigma, dilution 1∶200; mouse monoclonal anti-Glial fibrillary acidic protein (GFAP), G-3893 (Clone G-A-5), Sigma, dilution 1∶300; mouse monoclonal anti-neuronal class III β–tubulin (Tuj1), MMS-435P, Covance, dilution 1∶500, and rabbit anti-active caspase-3 (CASP3), C8487, Sigma, dilution 1∶300. Sections were incubated overnight at 4°C in the primary antibodies and then washed three times for 5 minutes each in Tris-buffered saline with 0.05% Tween-20. The bound primary antibodies were detected using biotinylated secondary antibodies (dilution 1∶200, Vector Laboratories Inc. and Jackson ImmunoResearch Laboratories Inc.) for 2 hours at room temperature. After three washes, sections were incubated for 1 hour at room temperature with streptavidin conjugated with fluorescein, Alexa Fluor 488 or horseradish peroxidase (HRP, dilution: 1∶200, Vector Laboratories Inc. and Jackson ImmunoResearch Laboratories Inc.). The HRP-linked secondary antibody was detected using 3,3′-diaminobenzidine (DAB, Sigma) as substrate with or without nickel chloride enhancement to be visualized as grey-black or brown specific stain deposits, respectively. After being washed three times, immunofluorescent staining brain sections were mounted in the presence of propidium iodide (PI, Sigma) to visualize cell nuclei. Primary antibodies were routinely omitted to determine the level of non-specific labeling. The sections were examined using a Nikon 80I microscope and an Olympus FluoView FV-1000 confocal microscope; figures were assembled in Adobe Photoshop 7.0.

### Immunoassayed cell counting

For quantification of NeuroD1-positive cells, sagittal slices of cerebella from P15 animals were processed for IHC and digitalized. In the analysis, the following two aspects were taken into consideration: cells migrating from the external to the internal granular layers (EGL and IGL, respectively), and the immunopositive resident cells in the IGL. Migrating cells were identified by their elongated shape and their association with vimentin-positive projections belonging to the Bergmann glia. Digitalization was standardized to 20× images of the cerebellar posterior lobes (VII-X). These lobes were selected because granule cell genesis and survival within them is highly dependent on the presence of NeuroD1 [30]; [34]. For statistical analysis, a total of 5 images per animal were quantified using a square of 1×10^−2^ mm^2^ which was applied as many times as the image allowed. For quantification of Purkinje cells, cerebellar sagittal sections from the four groups were immunostained for GAD67 and digitalized at 20x. The total number of Purkinje cells was expressed in an area of 4×10^−2^ mm^2^. The percentage of abnormal cells showing nuclear and/or cytoplasmic alterations and/or changes in the axial orientation was also determined.

### Western Blot

Nuclear and cytoplasmic proteins fractions from frozen cerebella were separated using the CelLytic NuCLEAR Extraction Kit (Sigma) according to the manufacturer's protocol. Lysis and extraction buffers were supplemented with the reducing agent dithiothreitol (DTT; final concentration: 1 mM), protease inhibitor cocktail [diluted from 100× stock solution stored at −20°C, composition: 4-(2-Aminoethyl) benzenesulfonyl fluoride (AEBSF), Pepstatin A, Bestatin, Leupeptin, Aprotinin, and trans-Epoxysuccinyl-L-leucyl-amido(4-guanidino)-butane (E-64)], and 10 mM (final concentration) sodium fluoride phosphatase inhibitor. Protein concentrations were determined by the Bradford method [52] using bovine serum albumin as standard. Nuclear and cytoplasmic fractions were used to study NeuroD1 and GAD67 or Tuj1 levels, respectively. A pool of 15 pineal glands, and pancreas from adult rats were processed using the same extraction kit to compare their GAD67 band patterns with those of cerebellum and hippocampus at P15 (the WB shown in Fig. S3 was generated in the absence of NaF). Proteins (80 µg per lane for NeuroD1, 60 µg per lane for GAD67, 30–45 µg per lane for Tuj1) were separated on 10% sodium dodecyl sulfate (SDS)-polyacrylamide gels, transferred to PVDF membranes by electroblotting and incubated in blocking solution (10% w/v low-fat milk powder in wash buffer: PBS with 0.05% Tween-20) for 1 hour at room temperature. The membranes were then rinsed three times with wash buffer for 10 min each and incubated overnight at 4°C with the primary antiserum diluted in blocking solution (sc-1084, dilution 1∶5,000) or in wash buffer (ab26116, 1∶5000; MMS-435P, 1∶5,000; rabbit polyclonal anti-actin, A 2066, Sigma, dilution 1∶5,000; rabbit polyclonal anti-histone H3, 07-690, Upstate, dilution 1∶10,000). The membranes were incubated with the corresponding biotinylated secondary antiserum diluted in wash buffer (1∶50,000, Vector Laboratories Inc.) for 1 hour at room temperature; rinsed three times and incubated with HRP-streptavidin conjugate diluted in wash buffer (1∶50,000, Vector Laboratories Inc.) for 1 hour at room temperature. Protein bands were visualized with the LAS-4000 system (Fujifilm) in a chemiluminescent reaction using a 1∶1 mixture of solution 1 (20 mM Tris-HCl pH 8.5; 2.5 mM luminol, Sigma; 0.4 mM coumaric acid, Sigma) and solution 2 (10 mM Tris-HCl pH 8.5; 0.02% H_2_O_2_). Histone H3 [53] and actin were used as loading controls for nuclear and cytoplasmic extracts, respectively. The optical density (OD) of target protein bands was determined from the LAS scan files using MacBiophotonic Image J software. Final values were expressed as the ratio of NeuroD1/Histone H3 in the nuclear fraction, and GAD67 or Tuj1/Actin in the cytoplasmic fraction.

### Statistical Analysis

Data, expressed as mean ± SEM, were analyzed using PRISM 5 (GraphPad Software, Inc.). Statistical differences were determined by one-way ANOVA followed by Bonferroni's post-hoc tests. A value of p<0.05 was considered significant.

## Results

### Effects of Pc and/or HI on cerebellar glia

We have previously demonstrated that the neonatal rat hypoxia-ischemia experimental model used here causes modifications at the cellular and molecular levels in several brain areas. When intermittent hypoxia (Pc) was applied 24 hours before hypoxia-ischemia, some of those modifications were attenuated or absent [48]. In order to establish the effects of Pc by itself, in the present study we included a group of animals exposed to this treatment on P7, without further injury. The tissues were examined on P15, in the same way as the other groups.

**Figure 1:**
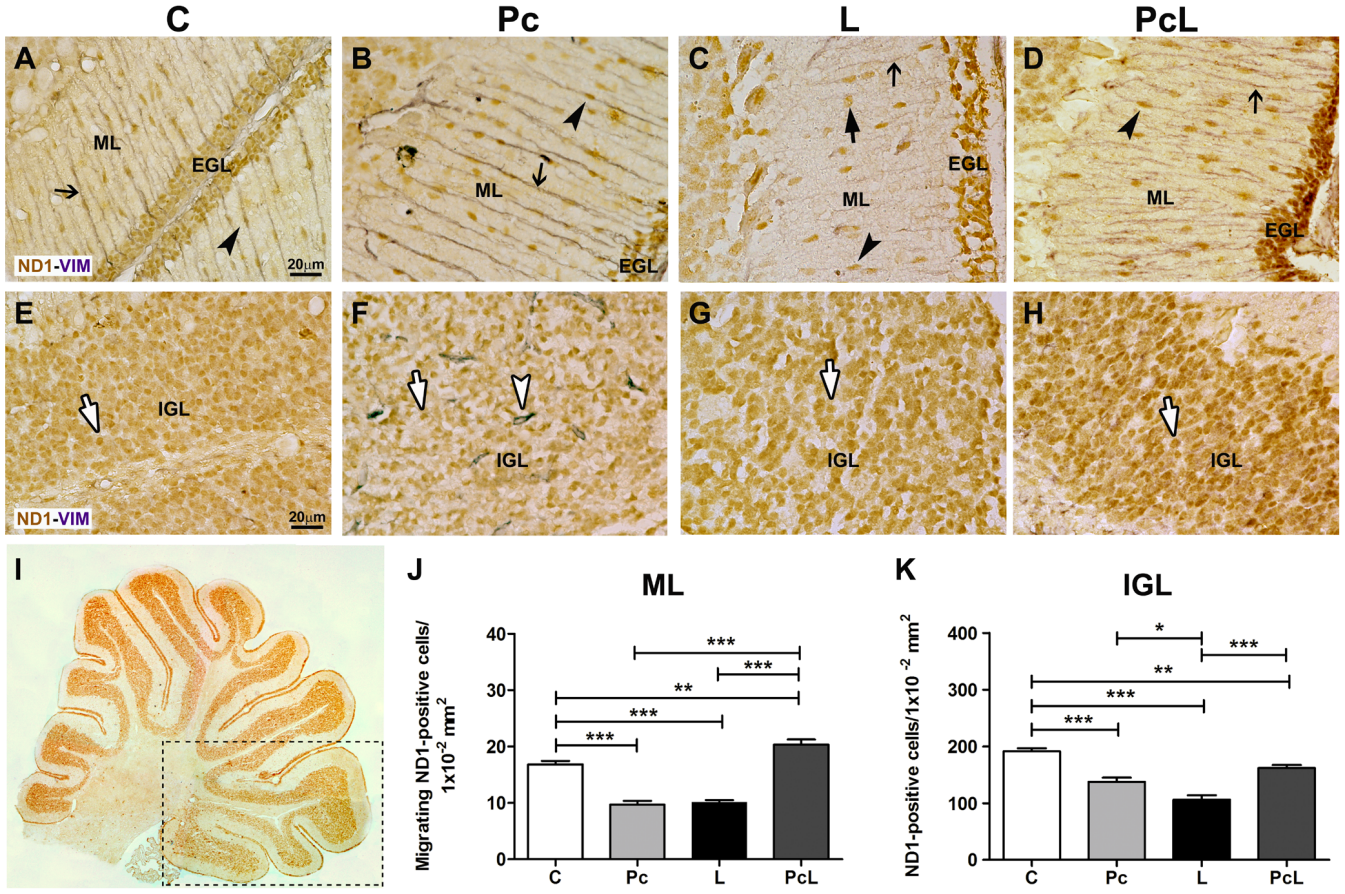
Effect of treatment conditions on NeuroD1 and vimentin-stained cells in the posterior cerebellum. Double immunolabeling for the pro-neuronal marker NeuroD1 (brown, HRP-DAB) and the glial intermediate filament protein vimentin (grey-black, HRP-DAB-nickel chloride) in the ML (A–D) and IGL (E–H) of control (C) and treated animals: hypoxic preconditioning (Pc), hypoxic-ischemic lesion (L) and injury with prior preconditioning (PcL). In A–D, migrating spindle-like NeuroD1-positive cells (black arrowheads) lying on the vimentin-positive Bergmann glial scaffolds (narrow black arrows) are shown. In panel C, fewer migrating cells are present in the ML of L animals, some of the NeuroD1-positive nuclei show a round profile (thick black arrow), while the vimentin labeling of the Bergmann glia projections is attenuated compared to the other three conditions. In D, an apparent recovery in the number of cells migrating on the vimentin-labeled Bergmann glia scaffold is observed in the PcL cerebellum. E-H: Images of the IGL from the same animals with intense NeuroD1 labeling of the granule cell nuclei (white arrows). Note the high number of granule cells in the C group (E) compared to the more scattered pattern in the Pc and L animals (F–G). In contrast, the PcL cerebellum shows an increased density of granule cells (H) compared to the individual treatments. Pc induces vascularization (white arrowhead, F). A–H: 60x, scale bar: 20 µm. I: Assembly of 4× images of the whole cerebellum immunolabeled with the anti-NeuroD1 antibody. The rectangle illustrates the posterior lobes where the quantifications of NeuroD1-positive cells in the ML (J) and in the IGL (K) were performed. Data are expressed as mean ± SEM in an area of 1×10^−2^ mm^2^. EGL: External granular layer. IGL: Internal granular layer. ML: Molecular layer. ND1: NeuroD1. VIM: Vimentin. Statistics: One-way ANOVA followed by Bonferroni's post-hoc tests; p<0.05; 0.01 and 0.001 (*, **, ***, respectively) were considered significant.

Initially, we evaluated the state of the radial glial scaffold in cerebellar tissue under control and experimental conditions, using an antibody for the intermediate filament (IF) marker vimentin which reacted with the Bergmann glia in all cases (Fig. 1 A–D). However, in the L group, the vimentin-stained glial processes were patchy and of irregular appearance, with discontinuous trajectories through the molecular layer (ML), in a region-dependent manner (Fig. 1 C and S2 D-D”). This alteration was not seen after preconditioning alone or in combination with L (Fig. 1 B and D), where the antibody revealed an ordered and regular layout of processes crossing from one side of the ML to the other without interruptions.

It is interesting to note that Pc animals displayed an increased expression of GFAP in cerebellar astrocytes compared to the normal expression in C animals, but with the same characteristics as the L and PcL groups (Fig. S1 A-D’ and S2). GFAP is highly expressed in the Bergmann glia scaffolds (Fig. S1 A–D and S2 C-C”), the structures that also contain vimentin intermediate filaments (Fig. 1 A–D and S2 D-D”). In addition, GFAP immunoreactivity in all three groups of hypoxiated animals revealed classical reactive astrocytes, with enlarged cell bodies and thickened branches populating the internal granular layer (IGL) and white matter (WM), indicating that the different treatments induced a long-lasting glial response with similar characteristics. These results imply that the cerebellar GFAP-positive astrocytes, unlike the Bergmann glia, are mainly sensitive to global hypoxia independent of the ischemic challenge. Thus, the cerebellar astrocytes marked with GFAP and/or vimentin in the treated animals appear heterogeneous in their response to the treatments, and the GFAP-positive cells are more abundant than the vimentin-positive ones, especially in the IGL (Fig. S2).

These regional and treatment-specific differential responses motivated us to explore the possibility of selective cerebellar cell damage, using one of the most common markers of apoptosis, activated caspase-3 (Fig. S1 E–H’). The results of this analysis were in agreement with previously reported developmental studies performed in the postnatal cerebellum [54]; [55], showing a profuse nuclear expression of activated caspase-3 in a cell stripe located adjacent to the Purkinje cell layer (PkL). We cannot positively identify these cells as either neurons or glia although, by their location and the size of the nuclei, many of them resemble the cell bodies of Bergmann glia. Interestingly, the expression pattern of these caspase-3-positive cells varies with the treatments: an apparently decreased number of labeled cells is observed in the Pc and PcL groups (Fig. S1 F-F’ and H-H’); while in the L group, which showed reduced vimentin staining, these cells seem to be spread over a wider area with a disorganized spatial configuration (Fig. S1 G and G’). In the L cerebellum, a high degree of nuclear vacuolation was also observed (Fig. S1 G’-inset). On the other hand, Pc and PcL groups display denser immunolabeling in the external granular layer (EGL) than the other control and L groups (Fig. S1 F-F’ and H-H’). We interpret these changes in caspase-3 expression as most likely being the result of modifications in the tissue milieu that may cause altered differentiating and migrating cell behavior [54]–[57], rather than as a sign of cell death.

### NeuroD1 expression in the cerebellum after hypoxia-ischemia and/or Pc

NeuroD1 plays an important role in the differentiation of specific neuronal cell types, including the cerebellar granule cells, where this bHLH factor was found to be necessary for normal development and adult function [30]; [34]; [35]. Because of this dual role, we were interested in investigating the possible influence of hypoxic Pc on cerebellar NeuroD1 expression in animals suffering neonatal brain injury.

Immunohistochemical analysis revealed that NeuroD1 is present in the nuclei of the P15 cerebellar granule cells of both the external (EGL) and internal (IGL) granular layers (Fig. 1 A–H). The staining pattern is consistent with reactivity to the whole population of precursors and differentiated granule cells in the EGL and IGL, respectively. In the molecular layer (ML), the labeled cells display the typical spindle-like appearance of migrating postmitotic cells that have departed from the EGL en route to the IGL, tightly associated with the guiding radial glial fibers. The NeuroD1-positive cells from the different treatment groups were counted in the cerebellar posterior lobes (Fig. 1 I). A significant decrease (p<0.001 vs. C) in the number of these cells was detected in the Pc and L groups in both the ML and IGL (Fig. 1 J and K). In the ML from L animals we also observed the presence of round-shaped cells attached to discontinuous vimentin-positive Bergmann glia processes described above (Fig. 1 C). We interpreted these as cells interrupted on their migratory route, which had recovered their original shape. Surprisingly, in the PcL cerebella, the number of NeuroD1-labeled cells increased significantly (p<0.01 vs. C) in the ML (Fig. 1 J). In the IGL from the same group an increase was also detected compared to the L animals (p<0.001), but was insufficient to recover to the number of granule cells present in the intact C cerebella (Fig. 1 K). This observation could be interpreted as a response of the tissue to the Pc stimulus, increasing the mobilization of cells from the EGL to the IGL, and ameliorating the depletion that is observed in the L group. The increased number of cells in the ML under PcL suggests that the recovery might continue, and reach or surpass control levels during the following days.

In the Pc animals, the expected increase in vimentin-labeling of the cerebellar microvessels was observed (Fig. 1 F), confirming the presence of global hypoxia-induced vascularization previously reported [58]; [59]. Intriguingly, in the cerebella of PcL animals, which had also undergone hypoxia-ischemia, this vascular reaction was not detected (Fig. 1 H).

**Figure 2:**
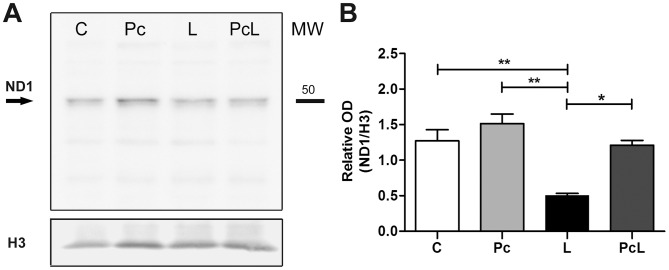
Cerebellar NeuroD1 levels under treatment conditions. Nuclear extract proteins from whole cerebella were analyzed for NeuroD1 and histone H3 levels via Western blot. A: Representative blot of three independent experiments using different litters from the four experimental groups showing a specific band for NeuroD1 of about 50 kDa (black arrow). The bands for the nuclear marker histone H3 for the same membrane are shown at the bottom. Quantifications expressed as the mean of the optical density (OD) of NeuroD1 relative to histone H3 ± SEM are graphed in B. H3: Histone H3. ND1: NeuroD1. MW: Molecular weight. Statistical analysis: One-way ANOVA followed by Bonferroni's post-hoc tests; p<0.05 and p<0.01 (* and **, respectively) were considered significant.

We also analyzed NeuroD1 levels in the four groups via Western blot (WB), using nuclear extracts from whole cerebella as a proxy for the transcriptionally active protein [27]; [28]. NeuroD1 was selectively diminished in the L animals compared with the rest of the groups (p<0.01 vs. C and Pc, p<0.05 vs. PcL), which showed no significant difference among them (Fig. 2 A and B). These results support the notion that Pc may prevent NeuroD1 inactivation when applied before hypoxia-ischemia.

### GAD67 expression in the cerebellum after hypoxia-ischemia and/or Pc

Cerebellar Purkinje cells have long been understood to be particularly sensitive to cerebral ischemia and excitotoxicity, an effect that has been attributed to the rapid loss of inhibitory GABAergic signaling caused by a decline in functional GABA_A_ receptors [60]. Alterations in GABA neurotransmitter signaling may affect the respiratory control centers in the brainstem and induce stress breathing responses commanded by neurons located in the cerebellar deep nuclei, thus influencing the ventilatory response of the body [61]. Indeed, Purkinje cells may play a general role in the modulation of respiration in consequence of their projections to the fastigial nucleus [62].

**Figure 3:**
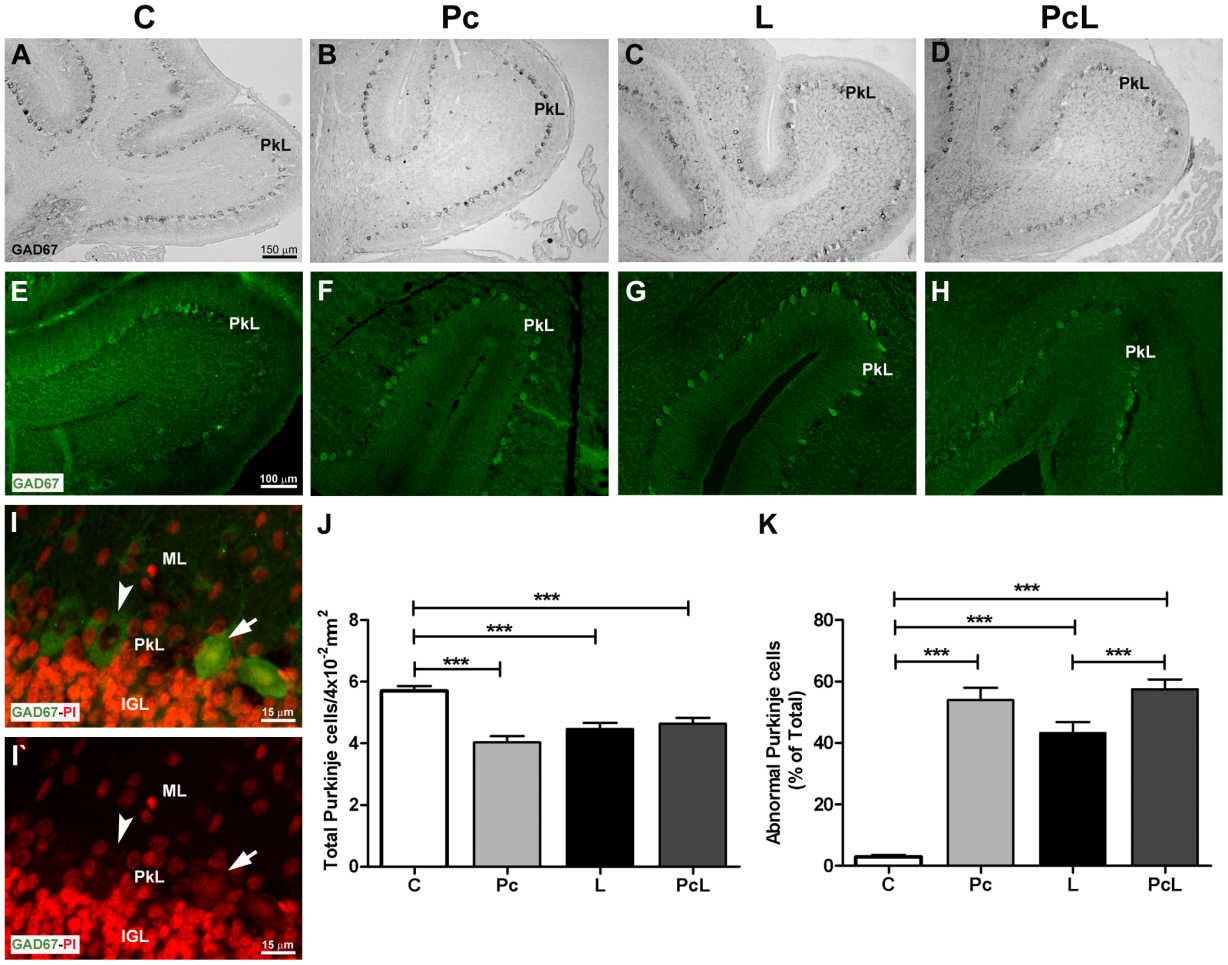
Treatment effects on cerebellar Purkinje cells revealed by GAD67 immunostaining. Posterior lobes of the four different groups immunolabeled for the GABAergic enzyme GAD67, and developed with HRP-DAB (A–D) and Alexa Fluor 488 (E–H) are shown. Both methods preferentially stain a string of large and evenly distributed cells in all treatment groups. Under control conditions (A and E), cell density and regularity in the PkL were highest. In the experimental animals that suffered hypoxia alone (Pc) or hypoxia and ischemia (L and PcL), the continuity of the string appears disrupted and the somas of some of these neurons show morphological modifications (B–D and F–H). In the three treated groups, at least two subpopulations of Purkinje cells were clearly distinguished that we categorized as normal (white arrowhead) or abnormal (white arrow) (I and I’ for the L group). In these enlarged images counterstained with propidium iodide (PI) to reveal nuclear compaction, the intensely stained nuclei of the abnormal cells and the mis-orientation of one of them with its long axis parallel to the ML and IGL are clearly seen. The quantification of the number of GAD67-positive Purkinje cells in the whole cerebella of the different groups of animals is shown in (J). The percentage of abnormal cells was also calculated and graphed (K). The data are expressed as the mean ± SEM in an area of 4×10^−2^ mm^2^. A-D: Optical microscopy, 10x, scale bar: 150 µm. E–H: Confocal microscopy, 20x, scale bar: 100 µm. I-I’: Epifluorescence microscopy, 2× zoom from 40x, scale bar: 15 µm. GAD67: Glutamate decarboxylase 67. PkL: Purkinje cell layer. Statistical analysis: One-way ANOVA with Bonferroni's post-hoc tests; (***) represents p<0.001 between the groups indicated by the horizontal bars on top.

For this reason, we looked at the effects of hypoxic-ischemic injury and/or Pc, on a GABAergic marker, GAD67, in the Purkinje cell layer (PkL) of the cerebellum (Fig. 3). In the whole cerebella of L animals we observed substantial changes in the number, morphology and spatial orientation of Purkinje cells. The regular assembly into a monolayer observed in normal tissue when these cells are labeled with an antibody specific for GAD67, was affected by the lesion (Fig. 3 C and G). Alternating with the typically-shaped Purkinje cells were others with altered features visible with propidium iodide (PI) staining; smaller intensely stained nuclei indicating high chromatin compaction, with indistinguishable nucleoli. These cells often appeared mis-oriented, with their long axis rotated parallel to the ML and IGL, replacing the perpendicular orientation characteristic of the normal cerebellum (Figure 3 I-I’). Remarkably, this pattern of altered Purkinje cells was present, with similar decreases (p<0.001 vs. C) in the total number of these cells, in all three experimental groups (Fig. 3 B–D, F–H and J). Moreover, the percentage of abnormal cells increased significantly (p<0.001) in the three hypoxiated groups when compared with C, but in the cerebella of the animals submitted to preconditioning treatment before hypoxia-ischemia, the PcL group, the abnormal cells significantly outnumbered (p<0.001) those without this treatment (L) (Fig. 3 K). We therefore hypothesize that these changes could be related to mechanisms of adaptation to the intermittent lack of oxygen during a crucial period of the cerebellar development [16]; [63].

**Figure 4:**
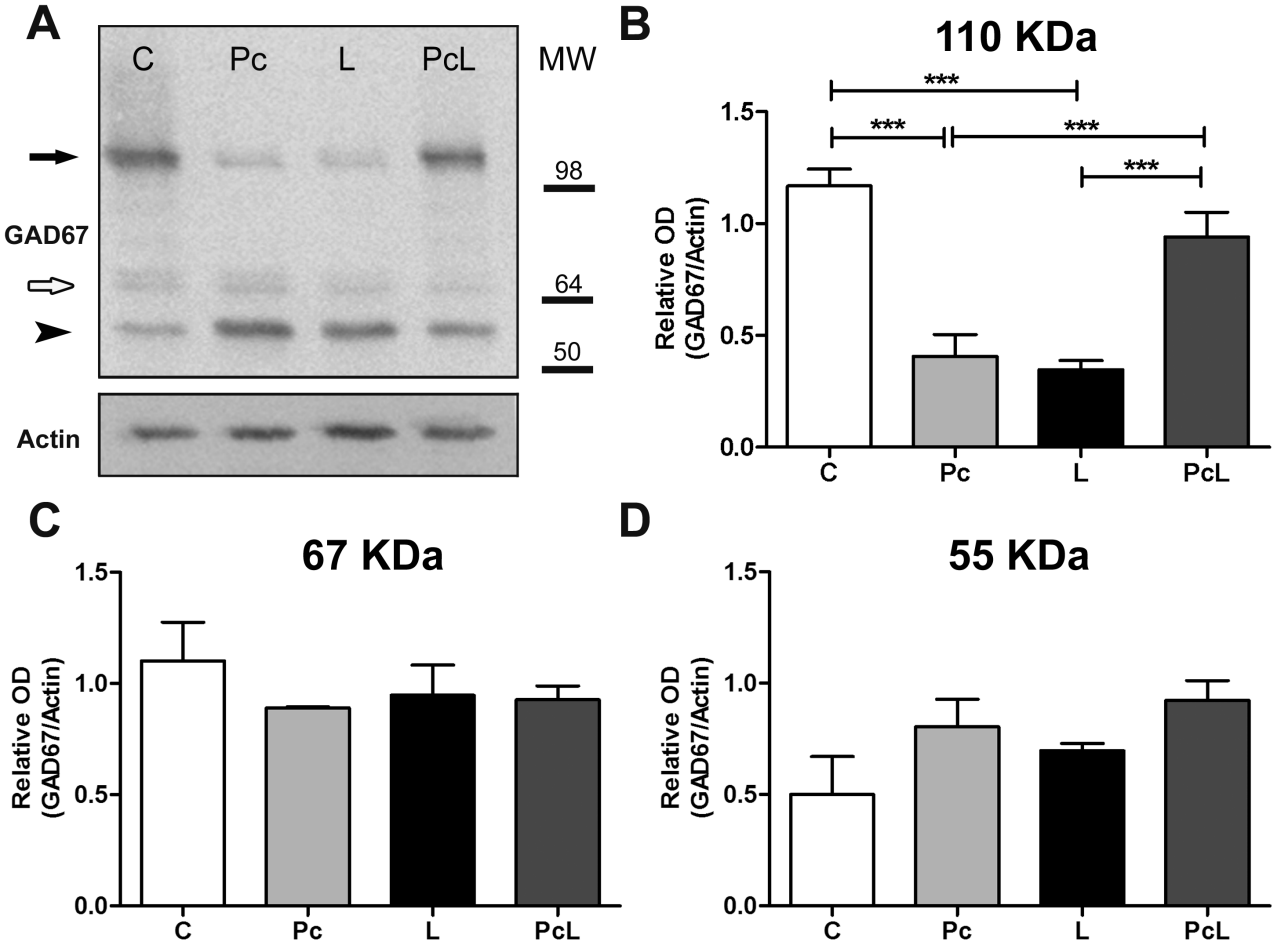
Total GAD67 protein expression in the rat cerebellum under treatment conditions. Levels of GAD67 in cytoplasmic extracts from whole cerebella were determined by WB and normalized for total actin in each sample. A: Western blot of one of three independent experiments using different litters from the four experimental groups. The black arrow points to a band of about 110 kDa, the white arrow points to a 67 kDa band, and the black arrowhead to a band of around 55 kDa (top). Actin was used as a control for protein loading (bottom). B–D: Quantification of the ∼110 kDa, 67 KDa and ∼55 kDa bands from the different experimental groups. Data were expressed as mean ± SEM. Statistical analysis: One-way ANOVA and Bonferroni's post-hoc tests. Comparisons between groups are shown by horizontal bars at the top; p<0.001 (***) was considered significant.

We then examined whether the reduction of GAD67-immunoreactive Purkinje cells reflected a decreased expression level of GAD67 after treatments, performing WB analysis of cytoplasmic extracts from whole cerebella (Fig. 4). To our surprise, the highly-specific monoclonal antibody consistently recognized bands other than the expected 67 kDa in our preparations. The ∼55 kDa band observed in our samples has previously been described in the enteric nervous system, and interpreted as a proteolytic cleavage product of GAD67 [64]. Higher molecular weight bands that represent multimers have previously been reported, including a band of approximately 110 kDa that corresponds to the homodimer of a truncated form of GAD67 [65]. The antibody used here does not bind GAD65, another isoform of the glutamate decarboxylase encoded by an independent gene (data confirmed by blasting the specific epitope) [29]. The three-band pattern observed in the cerebellum is similar to that seen in hippocampus (Fig. S3), but distinct from the pattern seen in endocrine tissue extracts, pineal gland and pancreas, which were enriched in the 67 kDa form while the other bands were faint or absent. Earlier reports have suggested that the major form of GAD67 in the rat cerebellum is multimeric [66]; [67], and that it undergoes calcium-dependent cleavage in rat synaptosomes [68]; [69]. We interpret our cerebellar staining pattern as showing canonical GAD67 as a minor form, with the major truncated form present in both monomeric (∼55 kDa) and dimeric (∼110 kDa) states.

The total amount of GAD67 distributed between the three immunoreactive forms in each sample (Fig. 4 A) and, individually, the levels of the ∼55 and 67 kDa forms did not change significantly after the treatments (Fig. 4 C and D), suggesting that these conditions do not induce the expression or cleavage of the coded 67 kDa form of GAD. Conversely, the relative optical density of the ∼110 kDa band decreased substantially (p<0.001 vs. C and PcL) after Pc and hypoxia-ischemia, the levels in the PcL and C groups not being significantly different (Fig. 4 B). While the experiment illustrated in Fig. 4 A suggests that some of the reduction in the amount of the ∼110 kDa form might be due to separation into the ∼55 kDa monomer, this was not consistently seen and not supported by the pooled data (Fig. 4 D). Thus the data suggest that approximately half of the dimerized form of GAD67 may be lost in the cerebellum of the neonatal rat after hypoxia alone or hypoxia-ischemia; but that this alteration is largely prevented when hypoxic Pc is applied before the ischemia. The functional significance of GAD67 dimerization is still debated, but may result in cellular compartmentalization, giving rise to the possibility that pre-synaptic GABA synthesis may be constrained after ischemia.

**Figure 5:**
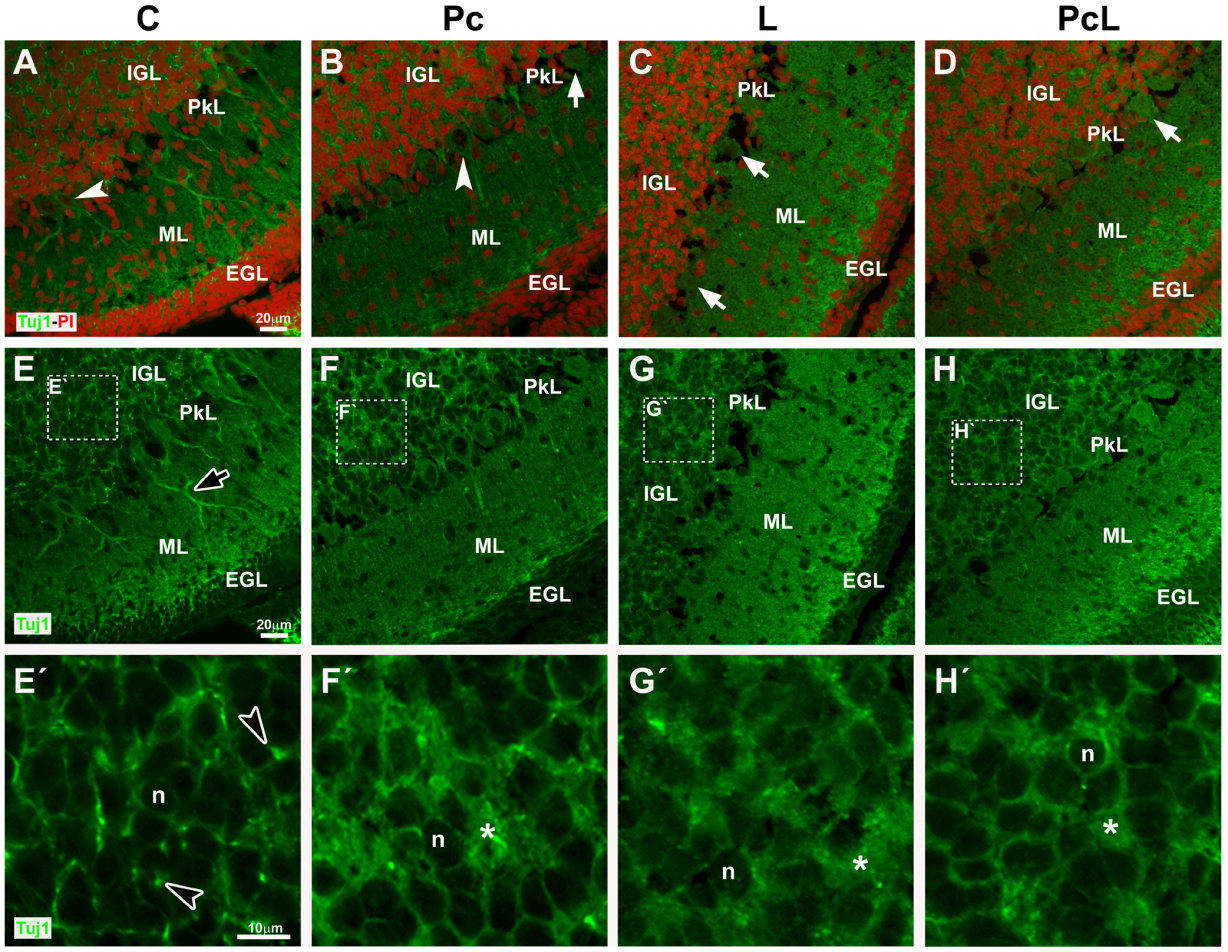
β–tubulin III (Tuj1) expression in cerebella under treatment conditions. A–D: Combined Tuj1 immunolabeling (green, Alexa Fluor 488) and PI staining of cell nuclei (red). Purkinje cells with normal and abnormal morphologies are indicated with white arrowheads and white arrows, respectively. E–H: The same fields viewed with the Tuj1 channel alone. Prominent dendritic trunks oriented towards the ML are observed in control animals (A and E, black arrow with white borders), but not under the treatment conditions. E’-H’: Enlargements of the insets shown in E–H. The IGL from control animals shows high cellular density with granule cell nuclei surrounded by narrow bands of cytoplasm and abundant highly immunoreactive spots (E’: black arrowheads with white borders). In the IGL from the Pc and L groups granule cells show a wider immunolabeled cytoplasm (F’-G’). In the PcL cerebellum the IGL has an intermediate appearance, between that of the untreated animals and the same layer in L and Pc rats (H’). n: Granule cell nuclei. *: Wider Tuj-1-positive cytoplasms. A-H: Confocal microscopy, 60x, scale bar: 20 µm. E’-H’: 4.5× digital zoom, scale bar: 10 µm.

GAD67 immunolabeling also allows the visualization of Purkinje cell dendritic arbors in the ML (Fig. 3 A and E). A noticeable reduction in GAD67 expression in the ML of L cerebella suggests that the normal growth of these cells at this stage of development, including dendritogenesis, may have been stunted (Fig. 3 G). Moreover, the nerve fiber marker β-tubulin III, also known as Tuj1, revealed a reduction in Purkinje cell arborization in the hypoxiated groups compared with C animals (Fig. 5 A–H). In addition, a lower Tuj1-positive cellular density in the IGL of Pc and L animals was observed (Fig. 5 B-F-F’ and C-G-G’), consistent with the previous observation of a reduction in NeuroD1-positive cells in this layer under those treatments (Fig. 1 K). Tuj1-positive somata of granule cells from C animals showed a narrow cytoplasm and the presence of abundant heavily immunostained spots (Fig. 5 E’). On the contrary, in the L and Pc animals the labeled area was wider together with a noticeable loss of densely stained spots (Fig. 5 F’ and G’). The PcL cerebellum showed a Tuj1 pattern intermediate between C and the other two groups (Fig. 5 H’). Analysis of Tuj1 levels in cytoplasmic extracts from whole cerebella by WB did not reveal any significant differences in total protein observed among the different groups (Fig. S4).

## Discussion

Birth asphyxia or intrapartum-related neonatal death is globally the 5^th^ most common cause of death among children under 5 years [70]. Survival may result in transient or permanent impairment of cerebellar functions, including motor control and cognitive and affective processes [71]; [72]. As there is at present an imperative need for devising preventive or palliative therapies for this condition, knowledge of the molecular mechanisms involved is of the utmost importance.

Here, we describe a series of disturbances occurring in the cerebellum between the first and second postnatal weeks, during one of the most important periods for the establishment of the fine–tuning of performance that include the formation of correct synaptic contacts. During the early postnatal period, the major source of input onto Purkinje cell somata switches from the glutamatergic climbing fibers (CF) to the GABAergic basket cell fibers (BF) [73]. From P9 to P15 there is a steep decrease in the CF-spine synapses in coincidence with the developmental elimination of perisomatic CF synapses, favouring a monopolized dendritic innervation by a single winner CF [74]. Perisomatic CF synapses peaked at P9, when pericellular nests were most developed and CF-spine synapses constituted 88% of the total perisomatic synapses. Thereafter, they diminished substantially by P15 and had almost disappeared by P20, whereas BF synapses increased reciprocally. Thus, changes in the density and composition of perisomatic CF synapses correspond to a developmental switch in the CF innervation mode; namely, CF-spine synapses increase during the pericellular nest stage, decrease during the capuchon stage, and are effectively gone in the dendritic stage. During the early postnatal period transmitter receptor expression on the Purkinje cell somata also switches from glutamatergic to GABAergic [75].

Our work documents varied responses to hypoxic and hypoxic-ischemic stimuli, involving cerebellar neurons of glutamatergic and GABAergic nature. These responses are summarized as follows: a decreased number of migrating and resident NeuroD1-positive granule cells in the ML and IGL, respectively, of the posterior cerebellar lobes from animals that suffered hypoxia (Pc) or hypoxia-ischemia (L), with an apparent recovery to control levels when the treatments were combined (PcL). Moreover, NeuroD1 protein expression in the nuclear fraction from the whole cerebellum (thus possibly including other cells expressing this factor) was affected by hypoxic-ischemic challenge, and again returned to control levels when the preconditioning stimulus was applied 24 hours prior. Interestingly, total NeuroD1 expression in Pc cerebella was not different from that in control pups.

GAD67-expressing Purkinje cells decreased in number in all three treatment groups, and a substantial percentage of the remaining cells evinced altered features, including diminished branching of their dendritic trees (visualized by Tuj1, a nerve fiber marker), pyknotic nuclei, and axial rotation. The expression of a potentially dimeric form of the truncated GAD67 protein in the cytoplasmic extracts from whole cerebella also fell in the Pc and L groups, while the PcL animals recovered to control levels. A decrease in Purkinje cell GAD67 mRNA expression was reported in autistic individuals, suggesting that reduced Purkinje cell GABA input to the cerebellar nuclei potentially disrupts cerebellar output to higher association cortices affecting motor and/or cognitive function [76]. It would not be unreasonable, therefore, to expect similar consequences after the hypoxia or hypoxia-ischemia reported in this study.

The tangential and radial migration of granule neurons from the surface of the cerebellar cortex (EGL) to their final destination in the deepest layer (IGL) is a complex process involving multiple intrinsic and extrinsic signals at defined points during development [22]. BDNF, EphrinB2/EphB2 and Slit/Robo signaling, among other pathways, control the movement of cells through attraction or repulsion. In addition, cytoskeletal proteins, such as actinomyosin, have also been described as important elements in this mechanism. Our results indicate that an hypoxic-ischemic challenge while these fine-tuning mechanisms are occurring apparently affects granule cell migration. Bergmann glia scaffolds seem intact in all experimental groups when labeled with the antibody to GFAP, but not when the antibody to vimentin was used. The L cerebella showed disrupted radial glia processes with an apparent pause in the motility of granule cells over the interrupted vimentin-immunolabeled guiding fibers. These results suggest a greater sensitivity of the vimentin-positive intermediate filaments (IF) to hypoxia-ischemia than the IF composed of GFAP. This differential response might reflect differences in the regulation of normal states and functions of these two cytoskeletal proteins, but it is noteworthy that the preconditioning stimulus prevents the alteration of the vimentin-positive IF caused by hypoxia-ischemia. Disruptions of cerebral radial glia after neonatal hypoxia-ischemia, and hypoxic preconditioning-induced precocious differentiation and maturation of astrocytes, have been described previously [77]; [78]. Furthermore, a role for vimentin in contributing to the normal development and morphology of the Bergmann glia, and its functional relationship with Purkinje cells, has been proposed by Colucci-Guyon et al. [79] on the basis of observations with mutant mice lacking vimentin. Interestingly, granule cell migration proceeded normally in this murine model. Based on our results, we cannot affirm whether or not disruption of vimentin IF in the radial glia of the L animals affected cerebellar granule cell migration, although a reduction of granule cells in the IGL was observed; a reduction that was not accompanied by substantial changes in the thickness of this layer. However, in view of the effects of hypoxic preconditioning on the promotion of a more mature form of intermediate filaments with higher expression of GFAP and S100β [78], the hypothesis that preconditioning causes an enhancement in the synthesis of certain proteins (in this case cytoskeletal proteins) involved in a metabolic reprogramming that shifts the threshold of damage is reinforced.

All the experimental conditions also increased activated astrocytes, mostly populating the white matter (WM) and the internal granular layer (IGL). Astrogliosis in L and PcL animals can be explained as a consequence of the massive inflammatory responses after the hypoxic-ischemic insult, affecting mainly cortical, striatal and thalamic regions of the brain, but extending to more distant regions such as cerebellum. However, a protective role of astrocytes after ischemia through multiple mechanisms has previously been posited [80]. The presence of reactive astrocytes in the animals receiving only the preconditioning stimulus could be explained by the role of these cells in reducing excess glutamate in the brain and so limiting excitotoxic cell death. This might in turn contribute to the hypoxic preconditioning-mediated neuroprotection, when applied 24 hours before the hypoxia-ischemia. Our results also indicate the presence of a heterogeneous population of reactive cerebellar astrocytes expressing GFAP and/or vimentin. The differential functions carried out by these two classes of intermediate filaments have been widely reviewed [81], and their dual role in both aggravating and repairing the injured tissue is beyond question.

Another complementary observation from our experiments supports the results of Oomman et al. [55] on the role of active (cleaved) caspase-3 in promoting differentiation of the Bergmann glia. Consistent with what these and other authors pointed out [56], we also report effects of the hypoxic-ischemic lesion on the Bergmann glia expressing active caspase-3, without compromising their survival. We assume that most of the cellular changes at the level of the Bergmann glia (enhancement of activated caspase-3 expression, cell misplacement, nuclear vacuolation) are transient, at least in the rat, and only present at the particular stage of development when this study was done, evidenced by the fast recovery and lack of sequelae observed in these animals when studied at a later period of development (results not shown). In addition, we observed that hypoxic preconditioning prevented Bergmann glia caspase-3 modifications caused by the lesion. It is possible that the Pc treatment may have interfered to some extent with non-cell death-related functions of the caspase-3 on these glial cells. Additionally, the key role of Bergmann glia in pruning CF synapses on Purkinje cells during the critical period from P9 to P20, when the wrapping of somatic spines by these neuroglial elements may suppress excessive or unnecessary receptor activation by keeping extracellular transmitter concentrations low around reorganizing perisomatic synapses [75], may also be compromised under hypoxia-ischemia.

In conclusion, we have shown that the developing cerebellum is also sensitive to global hypoxia and hypoxia-ischemia whose necrotic core is located primarily in the cerebral cortex, hippocampus and caudate-putamen 7 days after lesion (P15) in the rodent brain [82], and that various neuronal and glial populations are differentially affected. Some of the effects of this lesion can be partially attenuated by a preconditioning hypoxia. The glutamatergic neurons of the granule layer are pruned by hypoxia-ischemia but can recover when hypoxia is delivered as a preconditioning stimulus 24 hours prior to the lesion. On the other hand, the GABAergic population, and in particular a subset of Purkinje cells, is more susceptible to the injury and not responsive to the preconditioning treatment.

Our results support the concept of preconditioning as a stimulus that mobilizes cell type-specific mechanisms of endogenous repair or avoidance of injury, acting on numerous and diverse cell effectors including synaptic transmitters, enzymatic mediators, transcriptional factors and cytoskeletal components. These observations open a range of possibilities for exploring preventive strategies for the treatment of perinatal encephalopathy.

## Supporting Information

Figure S1 **Effects of hypoxic treatments on cerebellar tissue. Immunolabeling with GFAP antibody.** A-A’: The antibody recognizes astrocytes populating the IGL and WM (white arrow), and the radial Bergmann glia scaffold (black arrow) in the control animals. B-D, B’-D’: All treated groups show an increase of GFAP labeling throughout the cerebellum with abundant reactive astrocytes, with enlarged somas and thick branches (white arrowheads), in the same cerebellar areas as in the control rats. The Bergmann glia scaffolds seem normal (black arrows). A–D: 20x, scale bar: 50 µm. A’-D’: Enlargements of the insets shown in A–D, 60x, scale bar: 25 µm. **Immunolabeling for activated caspase-3.** E-H’: The immunostaining revealed a strongly reactive population of cells (black arrows) located in the proximity of the PkL. The number and distribution of these cells apparently varies among the different experimental groups. E–H: 20x, scale bar: 50 µm. E’-H’: Enlargements of the insets shown in E–H, 60x, scale bar: 25 µm Inset in G’: Vacuolation in immunoreactive nuclei. C: Control. Pc: Hypoxic preconditioning. L: Hypoxia-ischemia. PcL: Preconditioning plus hypoxia-ischemia. CASP3: Active caspase-3. EGL: External granular layer. GFAP: Glial fibrillar acidic protein. IGL: Internal granular layer. ML: Molecular layer. PkL: Purkinje cell layer. WM: White matter.
(TIF)

Figure S2 **Heterogeneity of astrocytes and Bergmann glia scaffolds in the hypoxic-ischemic (L) cerebellum.** A–F: Combined GFAP or vimentin immunolabeling (green, Alexa Fluor 488) and PI staining of cell nuclei (red) of consecutive sections from an injured (L) cerebellum. A’–F’: The same fields viewed with the IF marker channel alone. A” –F”: immunolabeling for IF developed with HRP-DAB-nickel chloride (grey-black). The astrocyte population is heterogeneous; GFAP-positive cells are more abundant than the vimentin-labeled ones. The vimentin-positive glial processes present discontinuous trajectories through the ML (white arrow) while GFAP-immunolabeled glial scaffolds seem ordered and regular (white arrowhead). A-F’: Epifluorescence microscopy. A” –F”: Optic microscopy. A-B”: 10x, scale bar: 150 µm. C-D”: 40x, scale bar: 50 µm. E-F”: 20x, scale bar: 100 µm. IF: Intermediate filament. PI: Propidium iodide. VIM: Vimentin.
(TIF)

Figure S3 **GAD67 expression pattern in nervous and endocrine tissues.** Cytoplasmic homogenates from P15 cerebellum and hippocampus, and adult pineal gland and pancreas were analyzed by Western blot. A clear pattern of three bands at apparent molecular weights of around 110, 67 and 55 kDa (black arrow, white arrow and black arrowhead, respectively), is observed in both cerebellum and hippocampus. In the glandular tissues, the 67 kDa band is the most prominent, while the other two bands are weak or absent. Molecular weight markers are indicated on the right. Cer: Cerebellum. Hip: Hippocampus. Pan: Pancreas. Pin: Pineal gland.
(TIF)

Figure S4 **Cerebellar Tuj1 levels under treatment conditions.** Tuj1 and actin levels proteins in whole cerebella were analyzed in cytoplasmic extracts by Western blot. A: Representative blot of three independent experiments using different litters from the four experimental groups showing a specific band for Tuj1 at about 50 kDa (black arrow). The bands for the protein loading control actin for the same membrane are shown at the bottom. Quantifications expressed as the mean of the optical density (OD) of Tuj1 relative to actin ± SEM are graphed in B. MW: Molecular weight. There were no statistically significant differences among the groups.
(TIF)
