# Supplementary figures and images for: Hypoxic Preconditioning Differentially Affects GABAergic and Glutamatergic Neuronal Cells in the Injured Cerebellum of the Neonatal Rat
Source: PLoS One. 2014 Jul 17;9(7):e102056. doi: 10.1371/journal.pone.0102056 (PMC4102512; doi:10.1371/journal.pone.0102056)

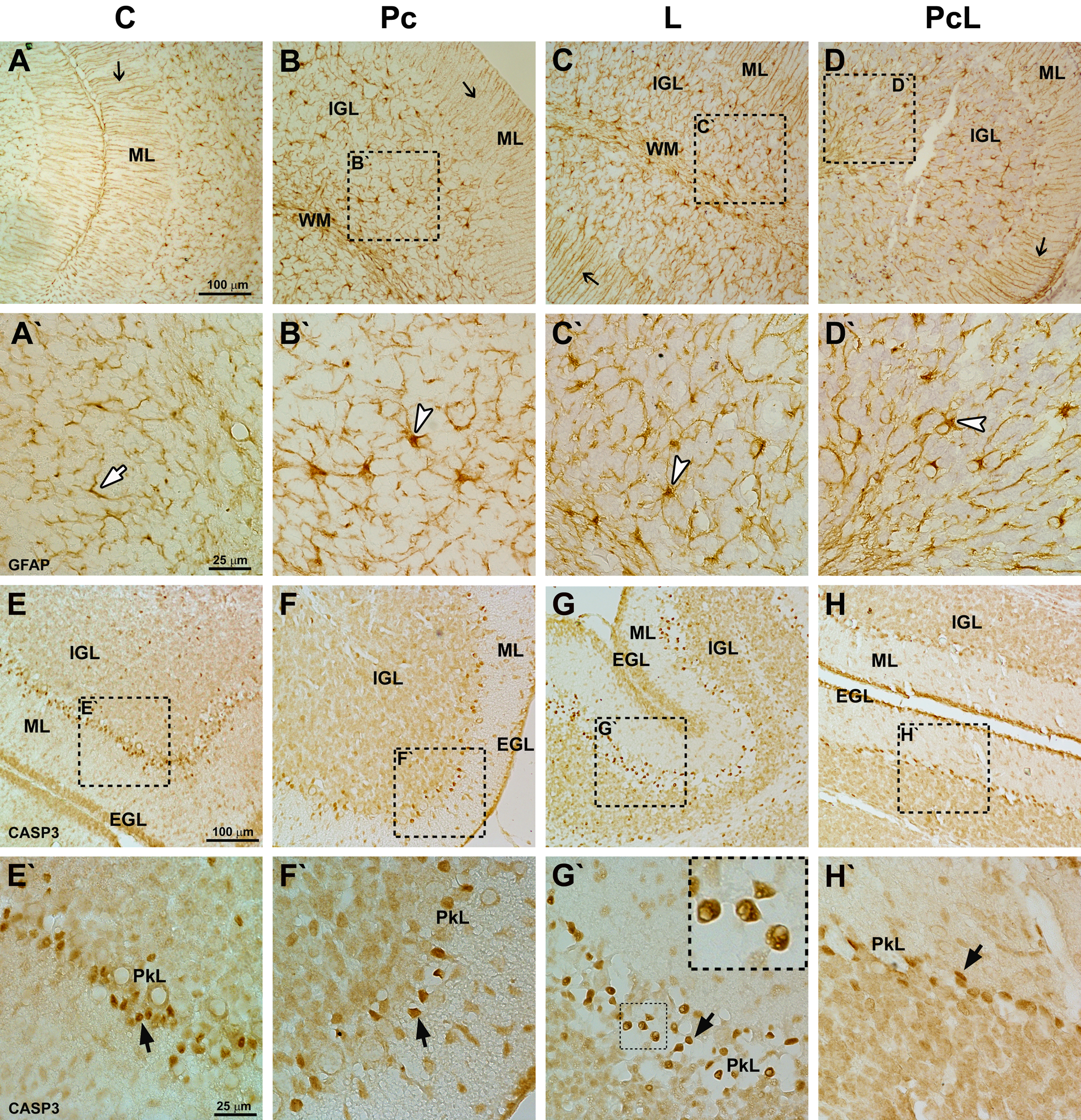

Supplement: Figure S1 — Effects of hypoxic treatments on cerebellar tissue. Immunolabeling with GFAP antibody. A-A’: The antibody recognizes astrocytes populating the IGL and WM (white arrow), and the radial Bergmann glia scaffold (black arrow) in the control animals. B-D, B’-D’: All treated groups show an increase of GFAP labeling throughout the cerebellum with abundant reactive astrocytes, with enlarged somas and thick branches (white arrowheads), in the same cerebellar areas as in the control rats. The Bergmann glia scaffolds seem normal (black arrows). A–D: 20x, scale bar: 50 µm. A’-D’: Enlargements of the insets shown in A–D, 60x, scale bar: 25 µm. Immunolabeling for activated caspase-3. E-H’: The immunostaining revealed a strongly reactive population of cells (black arrows) located in the proximity of the PkL. The number and distribution of these cells apparently varies among the different experimental groups. E–H: 20x, scale bar: 50 µm. E’-H’: Enlargements of the insets shown in E–H, 60x, scale bar: 25 µm Inset in G’: Vacuolation in immunoreactive nuclei. C: Control. Pc: Hypoxic preconditioning. L: Hypoxia-ischemia. PcL: Preconditioning plus hypoxia-ischemia. CASP3: Active caspase-3. EGL: External granular layer. GFAP: Glial fibrillar acidic protein. IGL: Internal granular layer. ML: Molecular layer. PkL: Purkinje cell layer. WM: White matter. (TIF) [file pone.0102056.s001.tif]

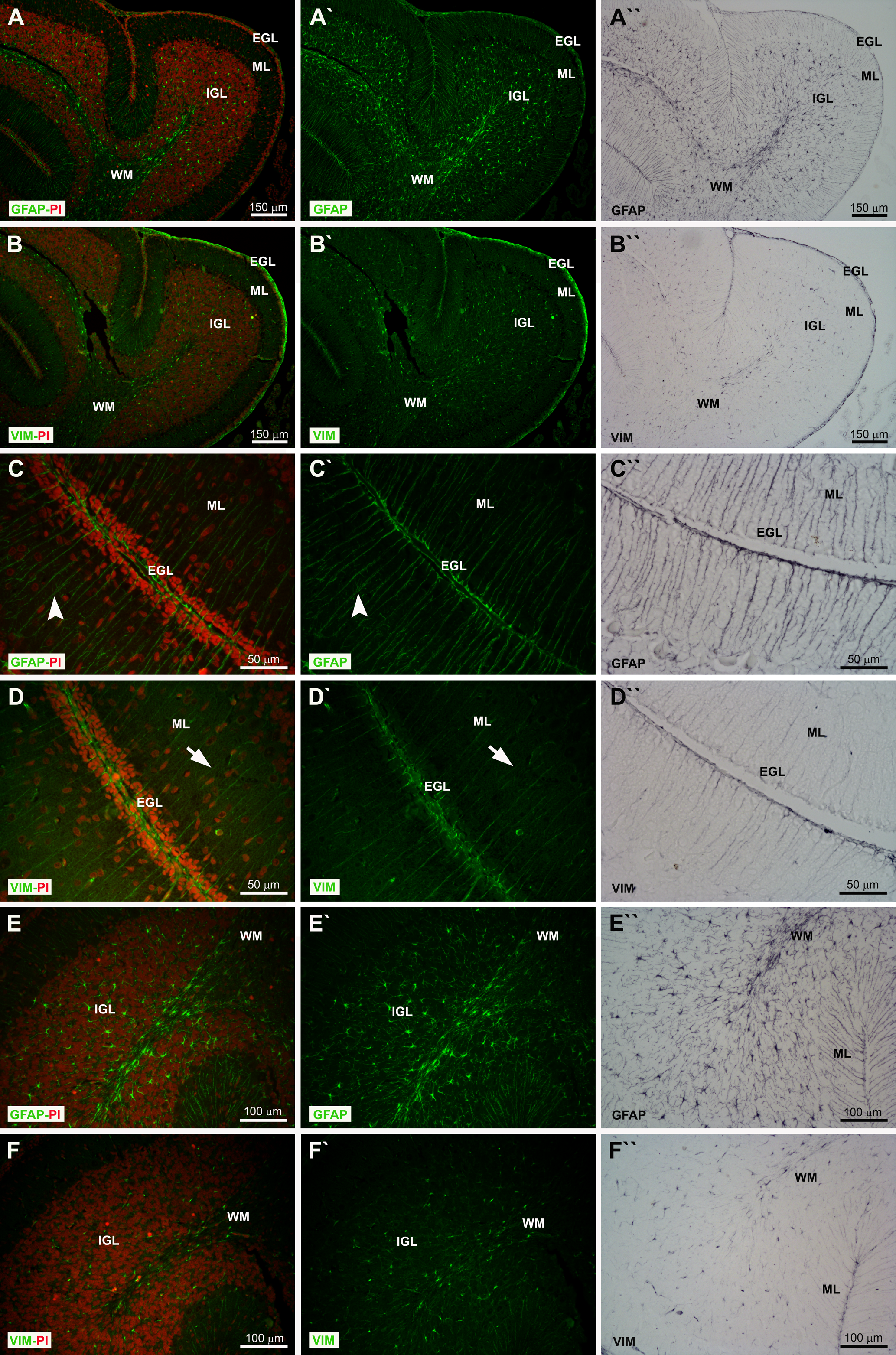

Supplement: Figure S2 — Heterogeneity of astrocytes and Bergmann glia scaffolds in the hypoxic-ischemic (L) cerebellum. A–F: Combined GFAP or vimentin immunolabeling (green, Alexa Fluor 488) and PI staining of cell nuclei (red) of consecutive sections from an injured (L) cerebellum. A’–F’: The same fields viewed with the IF marker channel alone. A” –F”: immunolabeling for IF developed with HRP-DAB-nickel chloride (grey-black). The astrocyte population is heterogeneous; GFAP-positive cells are more abundant than the vimentin-labeled ones. The vimentin-positive glial processes present discontinuous trajectories through the ML (white arrow) while GFAP-immunolabeled glial scaffolds seem ordered and regular (white arrowhead). A-F’: Epifluorescence microscopy. A” –F”: Optic microscopy. A-B”: 10x, scale bar: 150 µm. C-D”: 40x, scale bar: 50 µm. E-F”: 20x, scale bar: 100 µm. IF: Intermediate filament. PI: Propidium iodide. VIM: Vimentin. (TIF) [file pone.0102056.s002.tif]

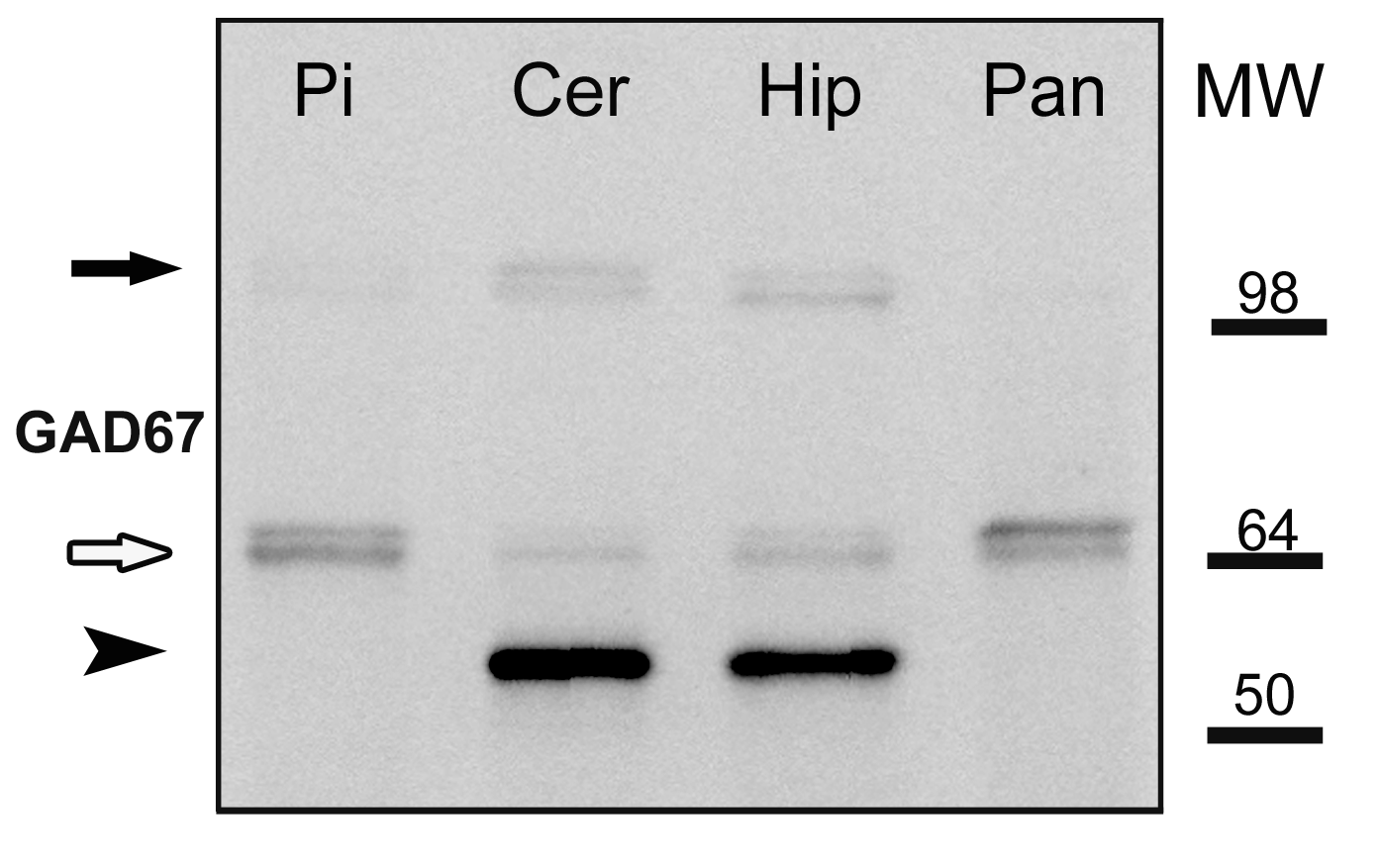

Supplement: Figure S3 — GAD67 expression pattern in nervous and endocrine tissues. Cytoplasmic homogenates from P15 cerebellum and hippocampus, and adult pineal gland and pancreas were analyzed by Western blot. A clear pattern of three bands at apparent molecular weights of around 110, 67 and 55 kDa (black arrow, white arrow and black arrowhead, respectively), is observed in both cerebellum and hippocampus. In the glandular tissues, the 67 kDa band is the most prominent, while the other two bands are weak or absent. Molecular weight markers are indicated on the right. Cer: Cerebellum. Hip: Hippocampus. Pan: Pancreas. Pin: Pineal gland. (TIF) [file pone.0102056.s003.tif]

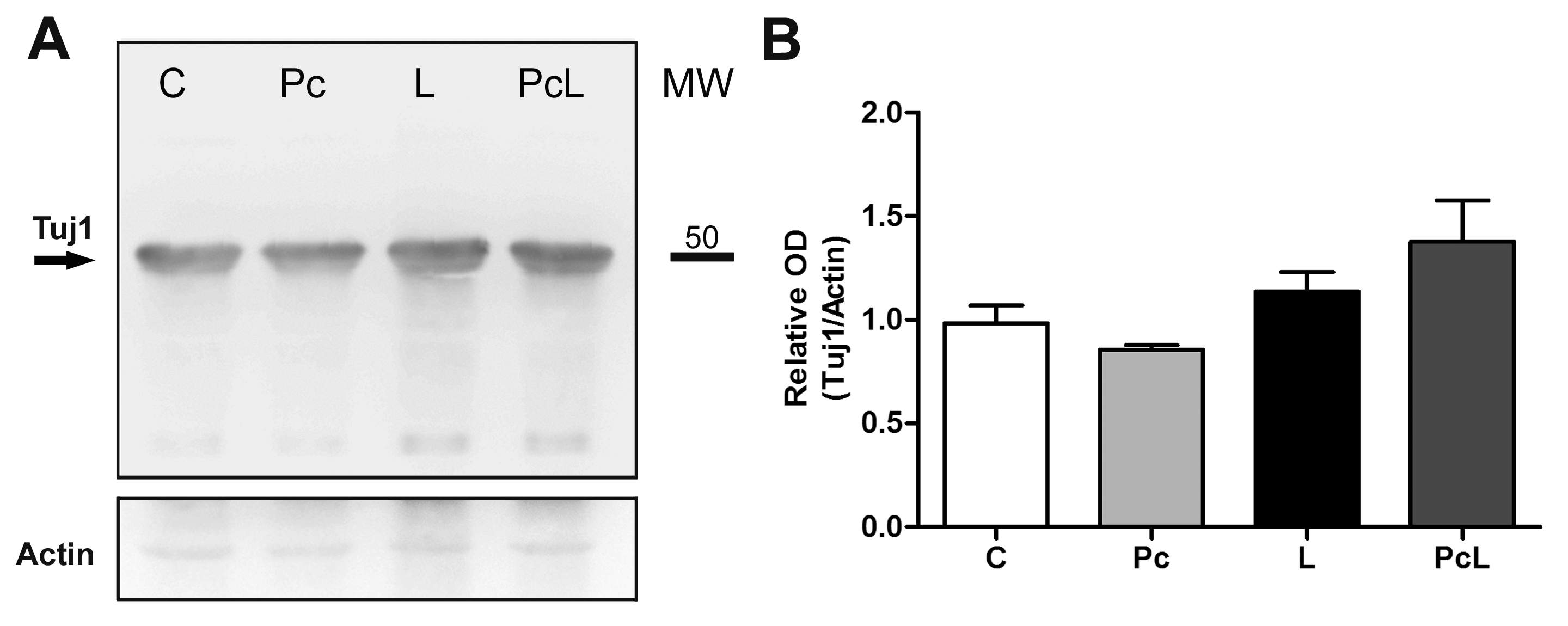

Supplement: Figure S4 — Cerebellar Tuj1 levels under treatment conditions. Tuj1 and actin levels proteins in whole cerebella were analyzed in cytoplasmic extracts by Western blot. A: Representative blot of three independent experiments using different litters from the four experimental groups showing a specific band for Tuj1 at about 50 kDa (black arrow). The bands for the protein loading control actin for the same membrane are shown at the bottom. Quantifications expressed as the mean of the optical density (OD) of Tuj1 relative to actin ± SEM are graphed in B. MW: Molecular weight. There were no statistically significant differences among the groups. (TIF) [file pone.0102056.s004.tif]
